# Numerical Study of Variation of Mechanical Properties of a Binary Aluminum Alloy with Respect to Its Grain Shapes ^†^

**DOI:** 10.3390/ma7043065

**Published:** 2014-04-15

**Authors:** Hamid Sharifi, Daniel Larouche

**Affiliations:** 1 Department of Mechanical Engineering, Laval University, Québec, QC, G1V 0A6, Canada; 2 Department of Mining, Metallurgy and Materials Engineering, Laval University, Québec, QC, G1V 0A6, Canada; E-Mail: daniel.larouche@gmn.ulaval.ca

**Keywords:** microstructure generation, multi-scale modeling, grain shapes, binary aluminum modeling, mechanical properties estimation

## Abstract

To study the variation of the mechanical behavior of binary aluminum copper alloys with respect to their microstructure, a numerical simulation of their granular structure was carried out. The microstructures are created by a repeated inclusion of some predefined basic grain shapes into a representative volume element until reaching a given volume percentage of the α-phase. Depending on the grain orientations, the coalescence of the grains can be performed. Different granular microstructures are created by using different basic grain shapes. Selecting a suitable set of basic grain shapes, the modeled microstructure exhibits a realistic aluminum alloy microstructure which can be adapted to a particular cooling condition. Our granular models are automatically converted to a finite element model. The effect of grain shapes and sizes on the variation of elastic modulus and plasticity of such a heterogeneous domain was investigated. Our results show that for a given α-phase fraction having different grain shapes and sizes, the elastic moduli and yield stresses are almost the same but the ultimate stress and elongation are more affected. Besides, we realized that the distribution of the θ phases inside the α phases is more important than the grain shape itself.

## 1. Introduction

A longstanding principle states that there is a distinct and identifiable relation between the interior structure of a material and its properties [1]. The majority of engineering materials have polycrystalline and multiphase structures. An important scale which affects the mechanical properties and stress distribution inside a material is the grain microstructure of the material. If a volume of a material contains a large numbers of grains with random crystal lattice orientations, the material could present isotropic characteristics even if the individual crystal is anisotropic in both elastic and plastic behaviors. Even though the overall mechanical property of a material is considered as isotropic, the stress distribution inside a volume of the material depends on its grain microstructure. The latter will have an impact on the global behavior of the material since stress concentration occurring at the microscopic scale initiates decohesion and global rupture eventually.

Several authors have worked on the development of microstructure models for the simulation of recrystallization and grain growth problems. These models can be divided into two groups [2]. The geometrical and topological model of the first group is based on combining the elementary geometry of nucleation, grain growth and impingement [3,4]. These models are mostly constructed by employing the Voronoi’s structure where the initial nucleation points are seeds in the Voronoi’s diagram. The second group, which is called component methods, is an extension of the first group to include several components like for example the grain orientations [5,6]. Nucleation and growth conditions are defined for each component. Different texture components grow independently and the final microstructure is formed when growing grains impinged and prevent their further growth. The nuclei can be distributed initially or they can be added continuously. The component method can be used for a three-dimensional (3D) simulation too. Another interesting method for modeling microstructure evolution processes is the phase-field method. This method defines a microstructure as a whole using some field variables which are functions of space [7].

When an acceptable microstructure geometric with distinguishable solid phases has been produced, the overall mechanical properties of the material can be found. There exist different approaches from different scales that can be used to calculate such properties; some of them are reviewed by Ortiz and Phillips [8]. Atomistic simulation is a powerful method to evaluate the mechanical properties of materials. However, it has serious difficulties, among them the limitation to work with small sizes and application of boundary conditions. Higher-level methods used by several researchers are based on polycrystal plasticity [9–12]. Here finite element formulations are used to describe the plasticity in the various grains. Some two dimensional models were presented by Becker and McHugh *et al*. [11,12]. Beaudoin *et al*. [10] presented a three dimensional finite element model for crystal plasticity with a viscoplastic constitutive formulation. The polycrystal was constructed by three-dimensional cuboid grains which formed a 2 × 2 × 2 array. Each grain can have different orientation, constitutive response, and is discretized to finer finite elements. Using such an approach, localized orientation gradients in face-centered polycrystals and the evolution of nonuniform deformation zones within individual crystals were simulated.

In this paper, we investigate the structure-properties relationship for a binary aluminum copper alloy having 4.6 weight percentage (wt%) Cu. A new method for producing discretized microstructure of alloys is presented, which focuses on the generation of a realistic microstructure while avoiding the complex procedures normally required when precipitation and growth of solid phases are considered. The generated microstructures can mimic typical 2D microstructures if suitable basic grain shapes are used. The resulting microstructure is transformed automatically into a finite element mesh. Using the ABAQUS software package, we investigated the variation of the mechanical properties with respect to variation of the microstructure grain shapes and sizes.

## 2. Grain Generation

**Figure 1.**
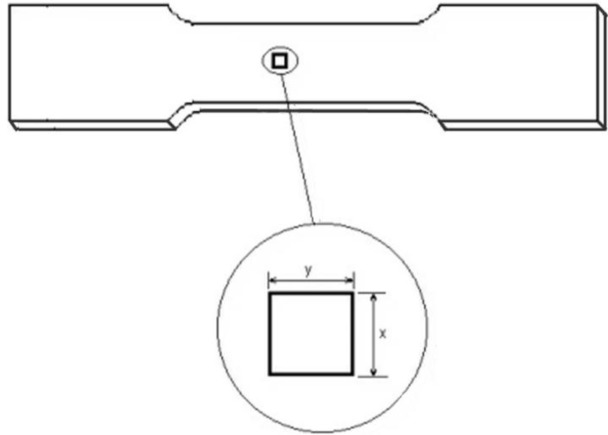
A tensile test specimen and a piece of such a specimen.

Let us consider a tensile test of a solid material using a thin specimen with a uniform thickness, as presented in Figure 1. The thickness of this specimen is considerably smaller than its other dimensions, so it is almost a plane stress problem. Consider a piece of such a test specimen which contains all the solid phases which have been formed during solidification or after the heat treating process. For example for an as-cast material this piece of material must be big enough to contain several solid grains and grain boundary phases, thus it is able to demonstrate the heterogeneous characteristic of the solid material satisfactorily. For simplicity, a rectangular domain having *x*, *y* dimensions has been chosen.

Our method is based on the creation of the grains inside this rectangular piece of material, which from now on will be called the representative volume element (RVE). As an example, if we consider the binary aluminum alloy having 4.6% copper, it is clear from the aluminum-copper phase diagram that the face centered cubic (FCC) phase α and the intermetallic phase θ (Al_2_Cu) can coexist in this alloy. Depending on the cooling conditions, each phase volume percentage can be calculated or determined by image analysis.

The general procedure is as follows: take a RVE completely fill with the θ phase, a basic grain shape is randomly chosen from a predefined basic grain shapes library, its orientation and its size are chosen randomly, and then it is placed in a random position inside the RVE. Some basic shapes will intersect each other inside the RVE and therefore some intersection rules, which depend on the grain orientation, are needed. The basic grains insertion is continued until a given volume fraction of α and θ phases is obtained, the latter being previously determined from a solidification model or from experimental measurements.

### 2.1. Basic Grain Shapes

**Figure 2.**
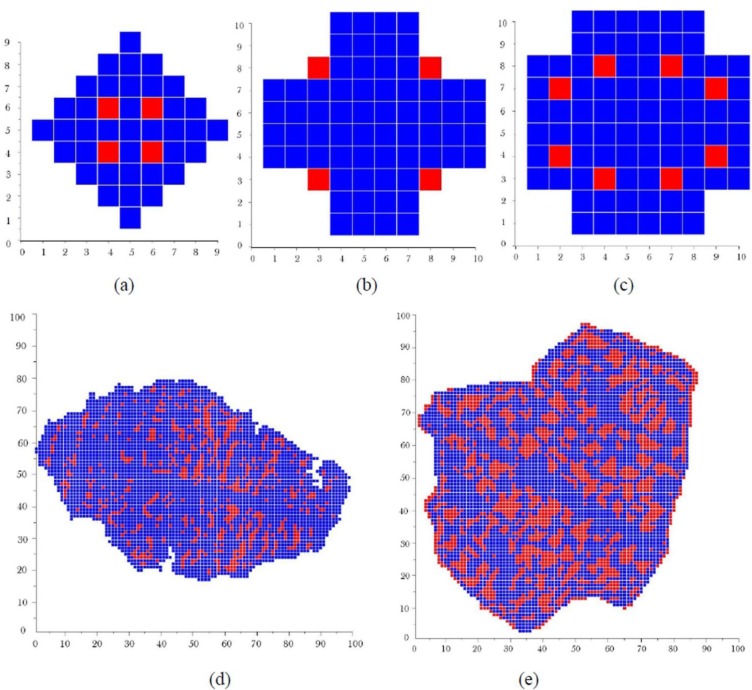
Some basic grain shapes. (**a**) grain shape *a* in a 9 × 9 elements array; (**b**) grain shape *b* in a 10 × 10 elements array; (**c**) grain shape *c* in 10 × 10 elements array ; (**d**) grain shape *d* in 100 × 100 elements array; (**e**) grain shape *e* in 100 × 100 elements array.

Figure 2a–c show some basic grain shapes used for a coarse representation of the grains. Here, each basic grain is constructed in an array of 10 × 10 or 9 × 9 elements. The blue squares in these figures represent the α phase elements while the red ones represent θ phase elements.

**Figure 3.**
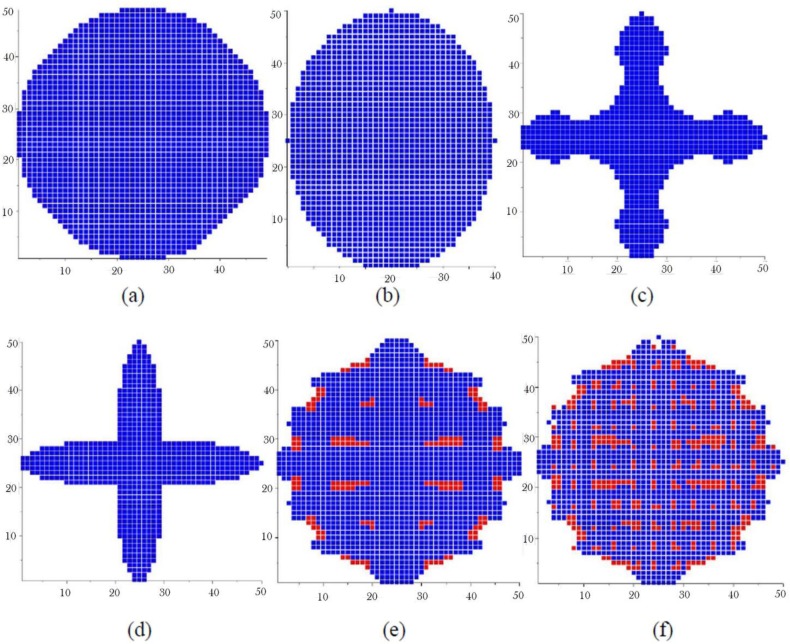
Basic grain shapes constructed in arrays of 50 × 50 elements. (**a**) grain shape 1; (**b**) grain shape 2; (**c**) grain shape 3; (**d**) grain shape 4; (**e**) grain shape 5; (**f**) grain shape 6.

The basic grains which are shown in Figure 2d,e are constructed in an array of 100 × 100 elements. They present some complex basic grain shapes that can be used for a fine representation of the basic grains. These shapes give more realistic grain microstructures since they were captured from a real micrograph. So, the quality of the final microstructure depends on the resolution of the basic grain shapes. The basic grains shown in Figure 3 have intermediate resolution since they are constructed in a 50 × 50 elements array. Taking into account the volume of computer memory needed and the speed of calculation, we mostly used 50 × 50 basic grain models for the grain generation and the finite element analysis. Not all of the basic grain shapes presented in Figure 3 produce realistic grain microstructures. However, one can use them to create different microstructures and to evaluate the effect of microstructure on the mechanical properties of a material.

### 2.2. Connection Criteria

After the creation of basic grain shapes, they must be projected into the RVE to obtain a granular structure. In the projection procedure, the size, the position and the orientation of each grain inside the RVE are chosen randomly. When a new grain occupies an area already covered by previous grains, a connection criterion is needed to decide whether these grains must be connected together or whether a new grain, with a θ phase joining band separator must be inserted.

**Figure 4.**
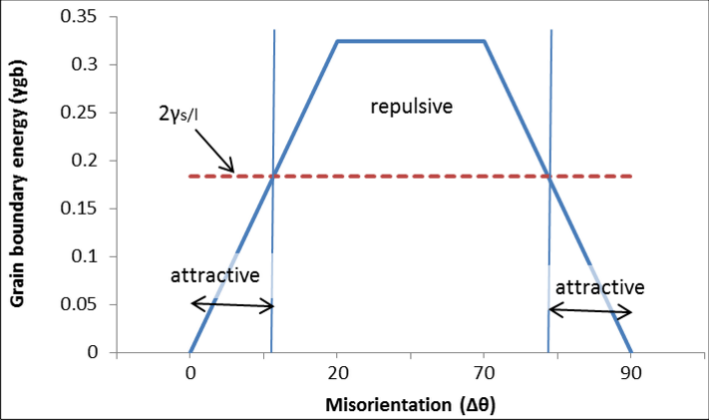
Grain boundary energy *vs*. misorientation (Δθ) in an aluminum alloy.

Hasson and Goux [13] considered the grain boundary energy (γ_gb_) as a function of the misorientation of the grains (Δθ) for an aluminum alloy. A simple approximation of this function was given by Mathier *et al*. [14], as presented in Figure 4. Rappaz *et al*. [15] considered the merging and the coalescence of two flat solid-liquid interfaces of unit area. They stated that the coalescence can be seen as the disappearance of two solid-liquid boundaries, each with energy γ_s/L_, and the formation of a solid-solid grain boundary with energy γ_gb_. Considering that γ_gb_ < 2γ_s/L_, then the free energy will be decreased if the solid-solid junction is formed. So it is an attractive situation. Looking at Figure 4, it can be seen that such a situation will happen for a low-orientation difference between two adjacent grains, namely if 0° ≤ Δθ ≤ 11° and 79° ≤ Δθ ≤ 90°. If γ_gb_ = 2γ_s/L_ then the free energy stays the same whether or not the solid-solid junction is formed. This refers to the neutral case. On the other hand, if γ_gb_ > 2γ_s/L_, then the free energy will be increased if the solid-solid junction is formed. So, it is a repulsive situation. Considering Figure 4, one concludes that such a situation can happen for high-orientation differences between two adjacent grains.

As a result, if a portion of the inserted grain is already occupied with the previous grain in the domain, the decision about the type of junction between the inserted grain and the old one will depend on the orientation difference between these two grains. If it is an attractive case, the grains must be connected and a single grain is formed. If it is a repulsive case, the old grain must be cut and the new grain is inserted in its position. Between the cut and the new grain, a θ phase joining band (channel) must be inserted to separate these grains. The existence of this phase at the intergranular position is to mimic the late solidification of the liquid phase existing between the grains where the liquid is rich in copper. Here, it is assumed that the liquid is forming a divorced eutectic microconstituent where the θ phase remains as a continuous film at the boundary.

**Figure 5.**
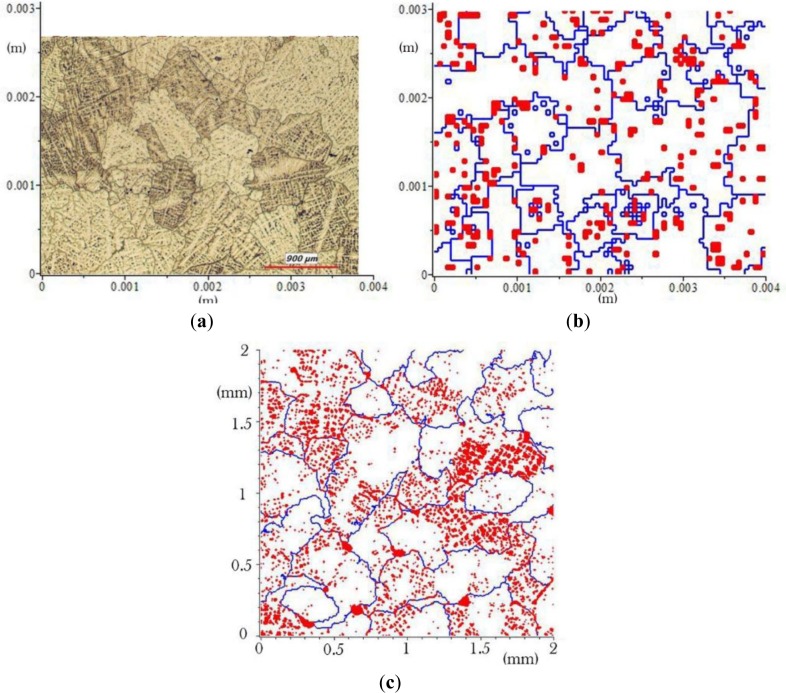
(**a**) Micrograph of an as-cast B206; (**b**) generated microstructure using basic grain shapes a, b and c of Figure 2; (**c**) generated microstructure using the basic grain shapes d and e.

Figure 5a shows the microstructure of an as-cast B206 aluminum alloy having approximately a volume fraction 95.6% of α phase and an average grain size of 550 μm. Black zones and lines are mostly θ phases inside the grains or between the grains. Figure 5b, shows the grains created for the same volume fraction of α phase in a 4 mm × 4 mm square RVE. The basic grain shapes a, b and c of Figure 2 with a resolution of 50 μm × 50 μm (size of the square elements composing the grain shapes) were used.

The blue lines are the joining θ phase channels generated by the grain connection procedure. The red squares are the θ phase elements that: (1) came with the basic grains and (2) remained between the grains once the insertion procedure finished. The comparison of this figure with Figure 5a shows that, even with simple basic grain shapes of low resolution, the microstructure of this alloy is simulated with a good approximation. Figure 5c presents the generated grains for the same alloy in a 2 mm × 2 mm square RVE with elements of 5 μm × 5 μm in size using the basic grain shapes c and d of Figure 2. As can be seen the generated microstructure is very similar to what can be seen in a real micrograph. This illustrates the power of our grain generation method to create complex 2D microstructures in a simple way.

**Figure 6.**
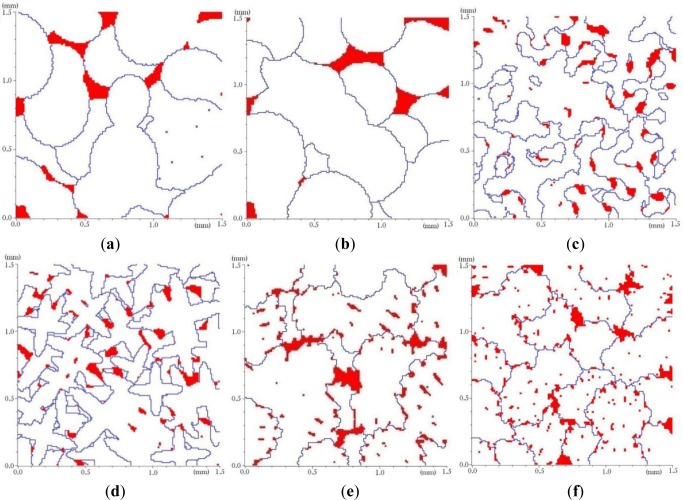
Generated microstructures using the basic grain shapes a to f of Figure 3. Volume fraction 95.6% of α phase and an average grain size of 550 μm were assumed for their generation. (a) basic grain a; (b) basic grain b; (c) basic grain c; (d) basic grain d; (e) basic grain e; (f) basic grain f.

Figure 6 shows microstructures having an intermediate resolution and constructed in a 1.5 mm × 1.5 mm square RVE with elements of 10 μm × 10 μm in size using the basic grain shapes presented in Figure 3, from a to f respectively. A volume fraction 95.6% of α phase is assumed for their generation. The average grain size is 550 μm. The microstructures obtained are quite different and not necessarily representative of a typical solidification process. Their generation can be used however to evaluate the impact of grain morphology on mechanical properties.

For microstructures presented in Figure 6e,f, a variable scale factor chosen randomly between 0.75 and 1.5 was applied to each basic grain before its insertion. This scaling range produces an average grain size that was fitted to the average grain size desired. To create microstructures having different average grain size, one can use the same basic grain shape but with a different scaling.

**Figure 7.**
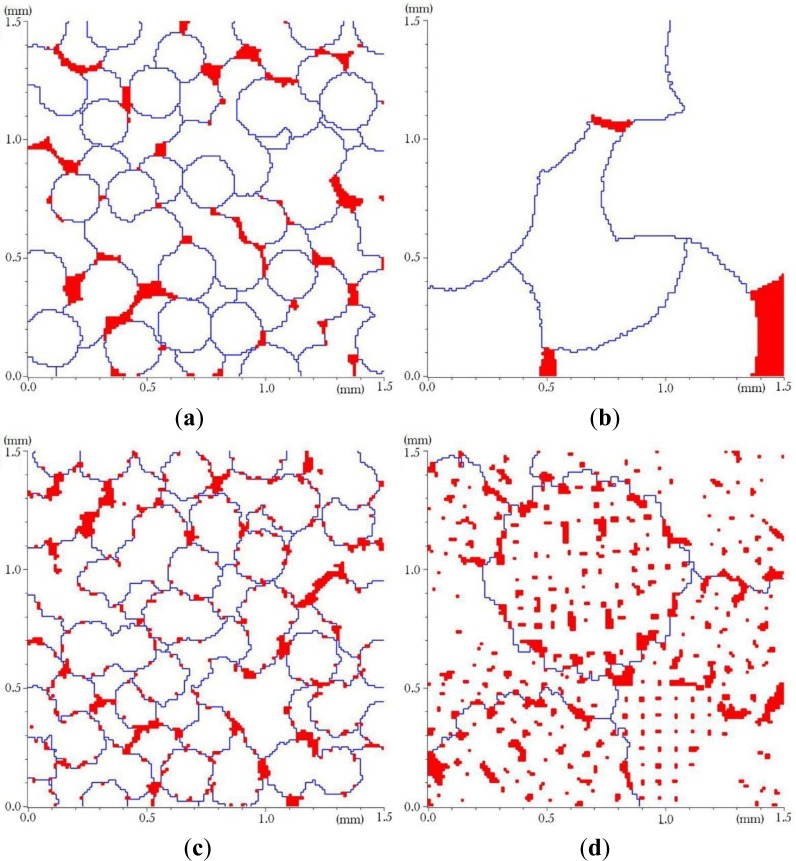
Generated microstructures, having a volume fraction 95.6% of α phase, using the basic grain shapes b and f of Figure 3: (**a**) 50% reduction of Figure 3b; (**b**) 200% size augmentation of Figure 3b; (**c**) 50% size reduction of Figure 3f; (**d**) 200% size augmentation of Figure 3f.

Figure 7 show some microstructures obtained with scaling factor of 50% and 200% of those used to generate the microstructures presented in Figure 6. For generation of microstructures with a half grain size of Figure 7a,c, a basic grain shape of Figure 3b,f with 50% size reduction has been used respectively. Figure 7b,d present microstructures generated from double size scaling of the basic grain of Figure 3b,f respectively. These microstructures were used to study the effect of grain size on the mechanical properties of alloys containing the same proportion of α and θ phases.

## 3. Thickness of the θ Phase Channels

Each generated grain structure is constructed for a given α to α + θ fraction determined according to the solidification path. One can divide the θ fraction in two parts; the first part contains the θ phase remaining between the grains after the insertion procedure and the θ phase inside the grains, which is already included in the basic grain shape. This subset of θ phase will be designated “pockets”. The second part contains the θ phases which exist in the form of channels between the grains made from a repulsive contact between the grains. This subset of θ phase will be designated “channel”. The volume fraction of these two subsets of θ phase can be written as follow:

$$F_{\theta }=F_{\theta \text{P}}+F_{\theta \text{C}}$$ (1)

Here, *F*_θ_ is the global fraction of θ phase, *F*_θP_ is the fraction of θ phase pockets and *F*_θC_ is the fraction of θ in the channels.

These values must be evaluated before starting the grain generation procedure. For instance, it is known that the volume fraction of channels depends on the cooling rate because of the back-diffusion phenomenon. So, an estimation of channel thicknesses is required to generate a suitable microstructure. At the beginning, the RVE is composed of N by N elements. A fraction *F*_θP_ of these elements will be θ phase at the end of the projection procedure. It is important to mention that channel elements will be added after this step. Since all elements have the same initial size at the projection procedure, one can write at this stage:

$$F_{\theta \text{P}}=\frac{\text{Number of θ elements}}{\text{Total number of elements in the control volume}}$$ (2)

The volume of θ phase inside the channels (*V*_θC_), is given by.

$$V_{\theta \text{C}}=F_{\theta \text{C}}\cdot V_{\text{RVE}}$$ (3)

where *V*_RVE_ is the volume of the RVE. Let *v*_i_ be the volume of a channel segment separating two adjacent elements, each belonging to different grains. The volume of channels must be equal to the sum of all these segments so one can write:

$$V_{\theta \text{C}}=\sum_{\text{i}}v_{\text{i}}$$ (4)

Channel segments may have different thicknesses (δ_i_). If *l*_elem_ is the length of the channel segment separating two adjacent elements, then we have:

$$v_{\text{i}}=\delta_{\text{i}}\cdot l_{\text{elem}}$$ (5)

For each pair of adjacent grains, a random channel thickness δ_i_ is assumed and is given as:

$$\delta_{\text{i}}=k\cdot r_{\text{i}}$$ (6)

where *r*_i_ is a random real number between 0.0 and 1.0 and *k* is a scaling factor, which must be determined to satisfy Equation (3).

As a result, one can write:

$$V_{\theta \text{C}}=\underset{\text{i}}{\sum }v_{\text{i}}=l_{\text{elem}}\cdot \underset{\text{i}}{\sum }\delta_{\text{i}}=l_{\text{elem}}\cdot k\cdot \underset{\text{i}}{\sum }r_{\text{i}}$$ (7)

Now, this equation can be solved for k.

$$k=\frac{V_{\theta \text{C}}}{l_{\text{elem}}}\cdot \sum_{i}r_{i}$$ (8)

## 4. Variation of the α Phase Properties

**Figure 8.**
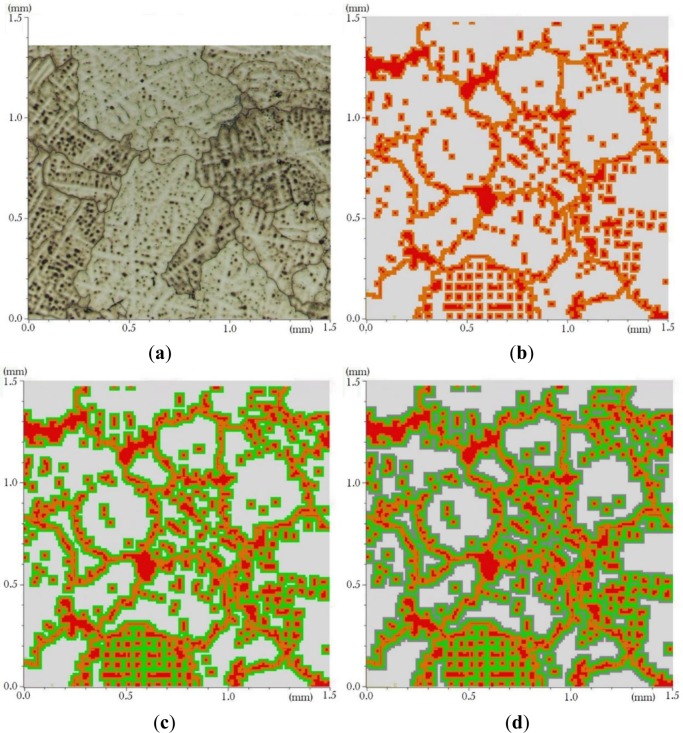
(**a**) As-cast microstructure of B206 alloy; (**b**) red regions are θ phase and orange regions are first layer α elements adjacent to the θ phase zones; (**c**) second layer is the green zones; (**d**) third layer is the grey zones.

For an as-cast Al-Cu alloy, the copper concentration varies from the center of a dendrite to its boundary because of the microsegregation phenomenon. Levasseur and Larouche [16] presented the concentration of copper by a Wave Dispersive X-ray Spectroscopy (WDS) line scan analysis performed on a columnar dendritic specimen of the binary Al-5.78 wt% Cu alloy. The sample was in as-cast condition and was produced with the directionally chilled casting procedure. Such a variation can even be seen in the microstructure photography. Figure 8a shows an as-cast B206 aluminum alloy microstructure where darker zones can be identified around the θ zones. This concentration profile produces a variation of the mechanical properties inside the α grains, particularly near the θ phases. To consider this type of variation a classification of elements was done and the α elements near to the θ phase elements were identified.

To classify α phase elements, the list of elements was searched and the elements having a θ phase neighbor were marked. This type of search can be repeated to mark the elements of the other neighboring layers. Different material properties could be assigned to the elements of each layer. Figure 8b shows θ phase and the first layer of neighboring α elements around the θ phase elements for a generated microstructure. Figure 8c,d present the second and the third layer of neighboring α respectively for the same microstructure.

## 5. Finite Element Mesh Generation

Creation of α phase finite elements are straightforward since each α element of the discrete domain can be presented as an α phase finite element. This is also true for the θ pocket zones as each θ element of the discrete domain can be converted to a θ phase finite element. The sole difficulty is the presentation of the θ phase channels.

**Figure 9.**
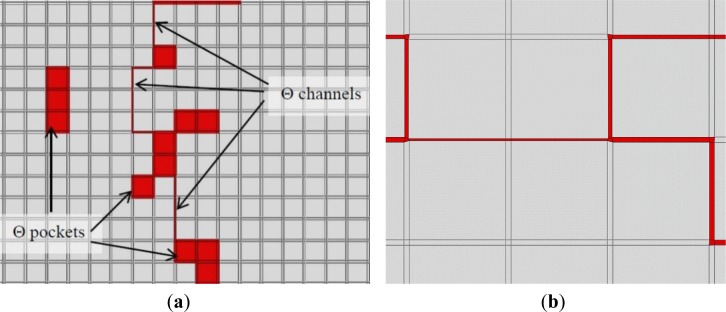
θ phase finite element generation; (**a**) θ phase channel and θ phase pocket elements; (**b**) θ phase channel elements with different thicknesses.

The θ phase channels are between two solid elements. The sizes of these channels, using calculations of the previous section, are already known. To create them, parts of the adjacent elements in both sides of the channel are taken and a θ phase finite element is created from these parts. To have a uniform finite elements mesh, half of the θ phase channel thickness is taken from each adjacent α elements. Notice that these elements belong to different grains so they can have different orientations. Figure 9a shows an example of the finite element generation in a portion of a generated microstructure.

The number of generated elements depends on the size and the resolution of RVE. It does not depend on the thickness of the channel since only one θ phase finite element is created between two grains. Figure 9b shows some θ phase finite elements with different channel thicknesses.

## 6. Results and Discussion

Using our grain generation procedure, different grain structures were created and converted in a finite element mesh. The mesh was thereafter introduced in the finite element software package ABAQUS. The Al-4.6% Cu was considered to have 4.4 volume percentage (vol%) of θ phase, among which, approximately 2.5 vol% was present in the channels generated by the connection procedure. The thickness of each channel, between two grains, was chosen randomly as mentioned in the previous section. Different loading and boundary conditions were applied. In these analyses, the α phase elements were considered as an elastoplastic material and the θ phase as an elastic material until its fracture point.

### 6.1. Finite Element Analysis

**Figure 10.**
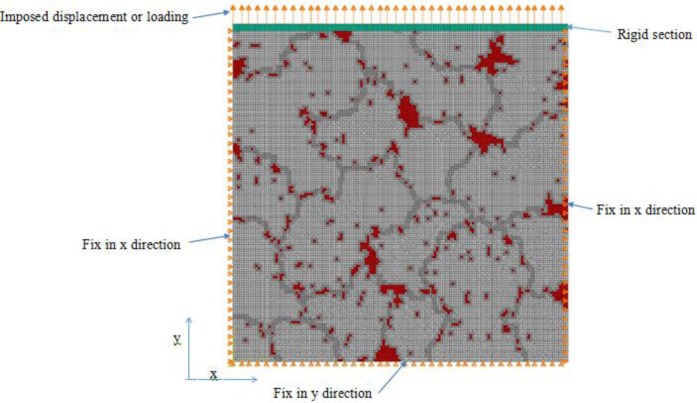
Finite element model of a 1.5 mm × 1.5 mm representative volume element (RVE). It contains 89,401 2D plain stress elements.

Figure 10 presents the generated finite element mesh and its boundary conditions applied on the 1.5 mm × 1.5 mm RVE presented in Figure 6f. It is composed of 89,401 2D plain stress elements. The elements of the first five rows from the top are considered rigid to be able to apply the tension load to the top of the domain. The bottom nodes are fixed in y direction and the side nodes are fixed in x direction. The elastoplastic material parameters implemented in ABAQUS are presented in Table 1.

As mentioned in previous sections, the copper weight percentage varies considerably near the θ phase. For an Al-4.6% Cu, the Cu concentration can vary from 2 wt% in the center of the dendrite to 5 wt% near the edge, so the material properties are expected to be not uniform in the α phase. It was therefore decided to give different material properties to the α phase elements depending on whether they were next to a θ phase or not. Table 1 presents the properties associated with the two types of phase α considered (near θ or not) and of the α phase itself. Notice that the α phase properties were taken from the B2219-T87 (Al-6.3 wt% Cu) and the B2117-T4 (Al-2.6 wt% Cu) alloys [17]. For θ-Al_2_Cu phase, the value of Young’s modulus reported by Eshelman and Smith [18] was used.

A variable displacement boundary condition was applied at the top of RVE. The imposed displacement started from zero and increased linearly until it reached its maximum values before the breakdown of the RVE. Using the *static general* step of the finite element analysis of ABAQUS software, two dimensional plane stress problems were solved. It takes only 6 minutes on a laptop computer having Intel(R) Core(TM) i7-3740QM CPU @ 2.7 GHz.

**Figure 11.**
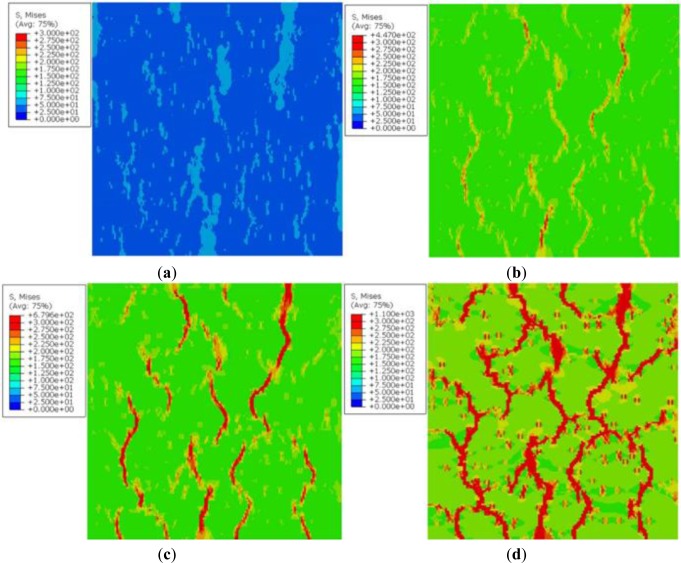
Von-Mises stresses inside the RVE of the microstructure of Figure 10 at different strains. (**a**) ε = 0.0006667; (**b**) ε = 0.003333; (**c**) ε = 0.005333; (**d**) ε = 0.02333.

As an example, Figure 11a–d present the Von-Mises stresses at different strains. These figures show how these stresses increased inside this heterogeneous material as tensile stretching occurs. One can see that the grain boundary zones are transferring a considerable amount of loading from top to bottom, if one compares Figure 11d with Figure 10.

**Figure 12.**
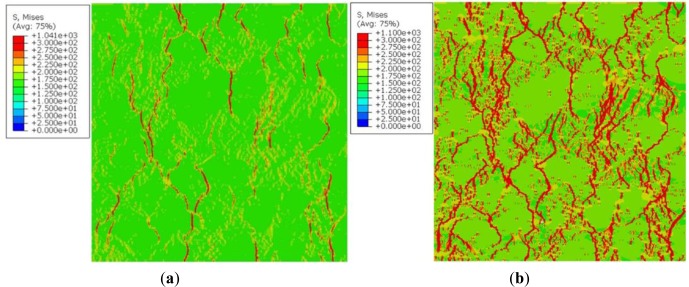
Von-Mises stresses inside the RVE of the microstructure of Figure 5c. (**a**) ε = 0.005; (**b**) ε = 0.023.

The distribution of the Von-Mises stresses at approximately the same imposed strain of Figure 11d, in a 1.5 mm × 1.5 mm RVE, are displayed in Figure 12b for the discretized microstructure presented in Figure 5c in a 2 mm ×2 mm RVE. The latter have a resolution of 5 μm instead of 10 μm in Figure 6f. As one can see, the stress patterns are similar though having better resolution stress contours.

### 6.2. Calculating Mechanical Properties of the Material

If one calculates the average stress as the sum of nodal reaction forces at the bottom of the RVE over the cross section area and the average strain as the displacement of the top nodes over the height of the RVE, the Young’s modulus can be found. For an elastic plane stress element the relation between stresses and strains are:

$$\left[\begin{array}{c} \sigma_{\text{xx}}\\ \sigma_{\text{yy}}\\ \tau_{\text{xy}} \end{array}\right]=\frac{E}{1-v^{2}}\left[\begin{array}{ccc} 1 & v & 0\\ v & 1 & 0\\ 0 & 0 & (1\;-v)/2 \end{array}\right]\left[\begin{array}{c} \varepsilon_{\text{xx}}\\ \varepsilon_{\text{yy}}\\ \gamma_{\text{xy}} \end{array}\right]$$ (9)

where *E* is the Young’s modulus and *v* is the Poisson’s ratio. Using our boundary conditions presented in Figure 10, it can be understood that ε_xx_=0, and γ_xy_=0. So, we have:

$$E=\frac{\sigma_{\text{yy}}}{\varepsilon_{\text{yy}}}(1-v^{2})$$ (10)

**Figure 13.**
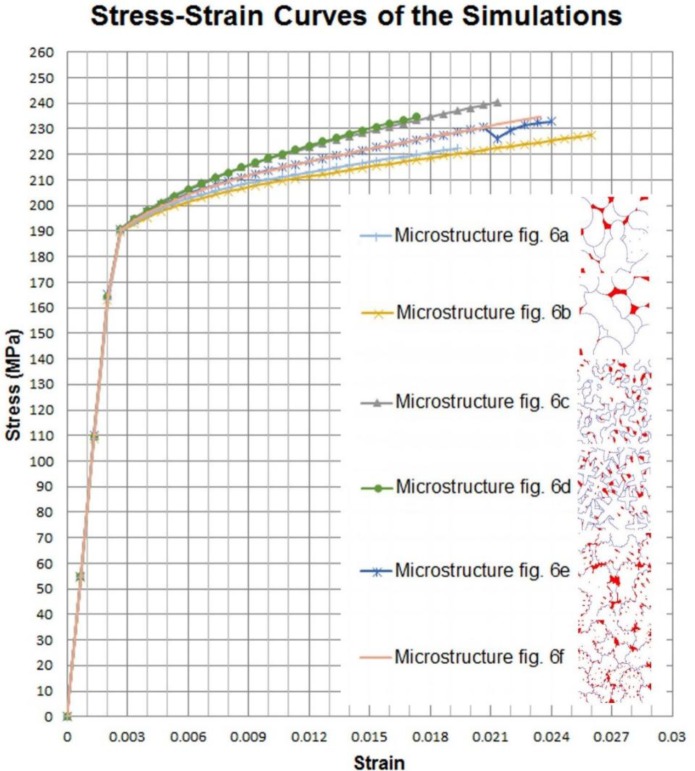
Overall stress-strain curves obtained with the discretized microstructures presented in Figure 6.

After the finite element execution, the ε_yy_ at the top of the RVE is known and the σ_yy_ can be evaluated as the sum of the reaction forces at the bottom of the RVE divided by the cross section area, so the Young’s modulus can be calculated and the stress-strain curve can be drawn. Those presented in Figure 13 were obtained with the discretized microstructures presented in Figure 6a–f.

Although the microstructures have obvious differences, one can see that they produce similar stress-strain curves from 0 to 190 MPa. So having the same volume fraction of α to θ phases, no matter the grain shapes, we have the same elastic behavior of the microstructure.

All the above models yield approximately at 165 MPa. The hardening parts of the stress-strain curves start to deviate after 190 MPa. Even after 190 MPa, we have some microstructures which behave similarly. The comparison of our simulation results with an as-cast B206 alloy (aluminum 4.6% copper) measurements is presented in Table 2. As can be seen from this table, our results of Young’s Modulus, Yield Stress and Ultimate Stress match very well with those of the as-cast B206 mechanical properties.

Three groups of microstructures can be identified from the above curves. Group A, which includes microstructures of Figure 6a,b, Group B, which includes microstructures of Figure 6c,d and group C, which includes microstructures of Figure 6e,f.

Considering Figure 6, one can see that the main difference between these groups comes from how θ phases are distributed inside α phases. In group A, θ phases are mostly concentrated between the grains, so the zones of α phases are large and soften the microstructure. In group B, many θ phase channels exists because of the fine dendritic arms of the grains, so they increase the hardening slope of the stress-strain curves. Group C is an intermediate case where we have a uniform distribution of θ phases inside more globular α grains.

**Figure 14.**
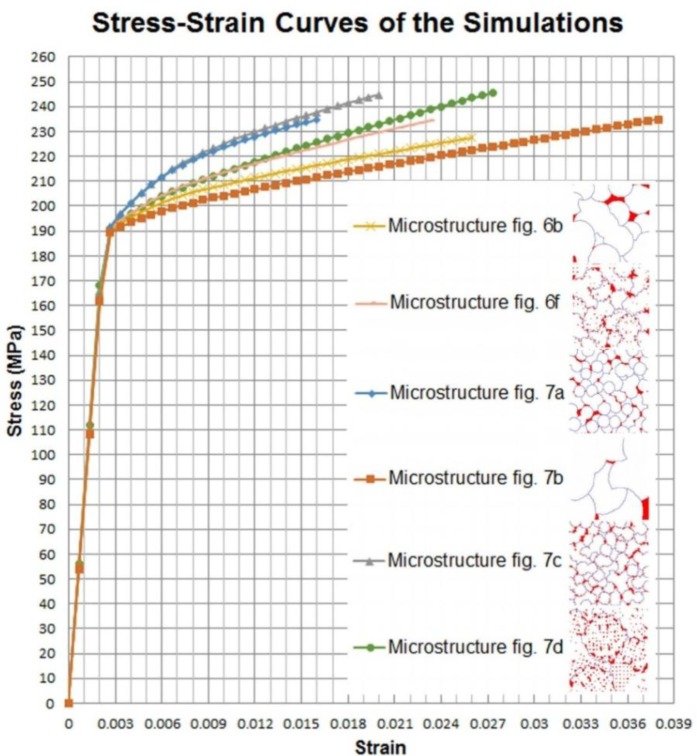
Overall stress-strain curves obtained with the discretized microstructures presented in Figures 6b,f and 7a–d.

To see the effect of the grain sizes on the mechanical properties of the alloy, microstructures of Figure 7a–d have been analysed with the same loading and boundary conditions. Figure 14 presents the stress-strain curves calculated.

In general, we can say that for the same θ and α phase fractions the smaller grain size promotes a higher hardening rate in the plastic regime. The effect of θ phase distribution must not be ignored since a microstructure having larger grains with a lot of θ phases between dendrite arms can present tougher properties. This is why the results of the model presented in Figures 6f and 7d are close to each other. It is worth pointing out that the distribution of θ phase had an immediate impact on the distribution of the α-near θ phase regions, so it is difficult to separate their effects. However, we found it important to include the α-near θ phase regions to simulate a more realistic mechanical behavior.

## 7. Conclusions

Using a simple numerical procedure, discretized microstructures were generated to mimic a realistic multiphase grain microstructure. A predefined basic grains database was used and a complex grain microstructure can be constructed with these shapes. The time consuming grain growth procedures were not used in this approach, which simplifies the method a lot. Zones of different phases can be formed between the generated grains depending of the connection procedure, the latter assuming the presence of intergranular secondary phases. The grain structure inside a given representative volume element is automatically transformed into a finite element mesh. Using a commercial finite element code, the mechanical properties of a binary aluminum 4.6% copper alloy having the same θ to α phase fraction but with different grain shapes and sizes were calculated. Our results show that even with different grain shapes and sizes, the elastic moduli and yield stresses are almost the same. We observe some difference in elongation, ultimate stresses and the slopes of the hardening part of stress-strain curves which indicates that grain shapes have a measurable influence on the plastic behavior of the alloys. According to these simulations, it seems that the distribution of the θ phase inside the α phase is more important than the grain shape. Note that, different distributions of the θ phase inside the α phase produce different stress fields inside the material.

Although, we have used this method for a binary aluminum alloy, it has the capacity to be used for the generation of other material microstructures. This model is also extendable to a 3D granular model.

## Figures and Tables

**Table 1.** Mechanical properties of the phases. The α phase properties were taken from the B2117-T4 (Al-2.6 wt% Cu) and the B2219-T87 (Al-6.3 wt% Cu) alloys [17]. For θ-Al_2_Cu phase, the value of Young’s modulus reported by Eshelman and Smith [18] was used.

Phase	α	α near θ	θ
Young’s modulus (GPa)	70	73	110
Poisson’s ratio	0.34	0.34	0.34
Yield strength (MPa)	165	395	≈1100
Ultimate strength (MPa)	295	475	–
Elongation (%)	24	10	–
Copper (%)	2.6	6.3	–
Other Elements (%)	0.35	0.36	–

**Table 2.** Comparison of properties obtained from different model microstructures with B206 alloy (as cast).

Alloy or Model	Young’s Modulus (GPa)	Yield Stress (MPa)	Ultimate Stress (MPa)	Elongation (%)
B206 as cast [19]	≈72	160–172	232–255	2.8–5
Figure 6a	72.1	163.0	222.3	1.9
Figure 6b	72.0	162.8	227.6	2.6
Figure 6c	72.7	164.4	240.5	2.1
Figure 6d	72.8	164.6	234.7	1.7
Figure 6e	73.1	165.2	233.0	2.4
Figure 6f	72.9	164.9	234.5	2.3

## References

[1] Phillips R. (1998). Multiscale modeling in the mechanics of materials. Curr. Opin. Solid State Mater. Sci.

[2] Raabe D. (1998). Computational Materials Science: The Simulation of Materials Microstructures and Properties.

[3] Mahin K.W., Hanson K., Morris J.W. (1980). Comparative analysis of the cellular and Johnson-Mehl microstructures through computer simulation. Acta Metall.

[4] Frost H.J., Whang J., Thompson C.V., Hansen N., Juul Jensen D., Leffers T., Ralph B. (1986). Modeling of grain growth in thin films.

[5] Juul J.D. (1992). Modelling of microstructure development during recrystallization. Scripta Metall. Mater.

[6] Juul J.D. (1997). Simulation of recrystallization microstructures and textures: Effects of preferential growth. Metall. Mater. Trans. A.

[7] Wang Y.Z., Chen L.Q., Kaufmann E.N., Abbaschian R., Bocarslyetal A. (1999). Simulation of Microstructure Evolution. Methods in Materials Research.

[8] Ortiz M., Phillips R. (1998). Nanomechanics of defects in solids. Adv. Appl. Mech.

[9] Dawson P.R., Marin E.B. (1998). Computational mechanics for metal deformation processes using polycrystal plasticity. Adv. Appl. Mech.

[10] Beaudoin A.J., Mecking H., Kocks U.F. (1996). Development of localized orientation gradients in FCC polycrystals. Philos. Mag. A.

[11] Becker R. (1991). Analysis of texture evolution in channel die compression. I. Effects of grain interaction. Acta Metall.

[12] McHugh P.E., Asaro R.J., Shih C.F. (1993). Computational modeling of metal matrix composite materials—I. Isothermal deformation patterns in ideal microstructures. Acta Metall.

[13] Hasson G.C., Goux C. (1971). Interfacial energies of tilt boundaries in aluminium: Experimental and theoretical determination. Scripta Metall.

[14] Mathier V., Jacot A., Rappaz M. (2004). Coalescence of equiaxed grains during solidification. Modell. Simul. Mater. Sci. Eng.

[15] Rappaz M., Jacot A., Boettinger W.J. (2003). Last stage solidification of alloys: A. Theoretical study of dendrite arm and grain. Coalescence. Metall. Mater. Trans. A.

[16] Levasseur D., Larouche D. (2011). Tensile creep testing of an Al–Cu alloy above solidus with a dynamic mechanical analyser. Mater. Sci. Eng. A.

[17] Kutz M. (2002). Handbook of Materials Selection.

[18] Eshelman F.R., Smith J.F. (1978). Singlecrystal elastic constants of Al2Cu. J. Appl. Phys.

[19] Kamga H.K., Larouche D., Bournane M., Rahem A. (2012). Mechanical properties of aluminium-copper B206 alloys with iron and silicon additions. Int. J. Cast Metals Res.

